# Study protocol for autism specific transition resources (T-Res Study): developing a flexible resource package for dealing with the loosening and/or lifting of COVID-19 related restrictions

**DOI:** 10.12688/hrbopenres.13155.1

**Published:** 2021-01-28

**Authors:** Sinéad Smyth, Nadine Mc Laughlin, Cillian Egan, Cathal Gurrin, Katie Quinn, Maria McGarrell, Sarah Devlin

**Affiliations:** 1 School of Psychology, Dublin City University, Glasnevin, Dublin 9, Ireland; 2 School of Computing, Dublin City University, Glasnevin, Dublin 9, Ireland; 3 As I Am, Rock House, Main Street Blackrock, Co. Dublin, Ireland; 4 St Ultan's Primary School, Cherry Orchard Avenue, Co. Dublin, Ireland; 5 National Council for Special Education, 1-2 Mill Street, Trim, Co. Meath., Ireland

**Keywords:** Autism, ASD, COVID-19, wellbeing, parenting, challenges, resources, transitions

## Abstract

Autism specific transition resources (T-Res) aims to develop a flexible resource package to support children and young people with a diagnosis of autism spectrum disorder (ASD), as well as their families and educators, during the loosening and/or lifting of coronavirus disease 2019 (COVID-19) related restrictions on movement. A secondary aim is to determine the current and long-term impacts of the COVID-19 related restrictions on the wellbeing of individuals with autism spectrum disorders and their parents/caregivers. Measuring and addressing the psychosocial impact of the COVID-19 pandemic and related restrictions in movement is of prime importance at this time.  The impacts of this crisis will be far reaching and many may not be realised for many years. The proposed research will focus on children and young people with a diagnosis of ASD, their families and educators.  The ASD population alone is sizable with 14,000 (or 1.55%) of students in schools holding a diagnosis. When parents, teachers, tutors and special needs assistants (SNAs) are also considered this is a considerable group. The proposed research has the potential to have impacts that are social, psychological, educational and economic. This will be achieved through development of an online transition package to guide parents and educators in preparing children and young people for the resumption of regular daily routines following the lifting of COVID-19 restrictions.  This resource will be developed based on the needs of families and young people, as measured through surveys, as well as expert consensus on the targets and means of intervention.  This ambitious project can be commenced quickly and is designed to produce outputs quickly, which will in turn be disseminated to key stakeholders.

## Introduction

### Autism spectrum disorder (ASD)

Autism spectrum disorder (ASD) is a chronic neurodevelopmental condition which manifests in early childhood and is marked by repetitive behaviour and deficits in social communication ( American Psychiatric Association, 2013). Current estimates place worldwide prevalence of ASD at 62 per 10,000 children ( Elsabbagh *et al.*, 2012). Irish figures estimate a prevalence rate of 1% ( Boilson *et al.*, 2016). Furthermore, according to the National Council for Special Education ( NCSE, 2016) almost 14,000 students in schools have been diagnosed with autism – this is 1 in every 65 students in schools (1.55% of all students). In addition to the core features of the disorder, research has demonstrated high levels of comorbid mental health problems in individuals with ASD.

### Coronavirus disease 2019 (COVID-19) and ASD

Individuals with a diagnosis of ASD are one vulnerable minority group which, like many others, has been negatively impacted by the COVID-19 related restrictions. Students with ASD typically receive additional educational supports focusing on academic, communicative, social and life skills. These are currently curtailed or unavailable. The impacts will be educational, psychological, behavioural and social impacts. Anxiety is a known comorbidity of ASD ( MacNeil *et al.*, 2009) and can be exacerbated by strange situations and changes in routine ( Bell *et al.*, 2017). Challenging behaviours may also be increased during times of transition ( Lequia *et al.*, 2012). School refusal is significantly higher in students with ASD than for neurotypical children ( Munkhaugen *et al.*, 2017) and an Irish survey found that 17% of respondents (N=556) did not regularly attend school due to school refusal, reduced school hours and that in a high proportion of cases this was due to anxiety ( AsIAm, 2019). It is highly likely that the period of COVID-19 restrictions which resulted in school closures from March 13 ^th^ 2020 until the end of the school year will have exacerbated this. This may result in more children refusing to attend school and requiring home tuition, which would result in a potentially added financial cost to the state in the long term. In addition to the potential impacts on children and young people with autism, it is highly likely that the parents and caregivers of these children have also been negatively impacted. Previous research has demonstrated that parenting stress is positively related to levels of challenging behaviour ( Kinnear *et al.*, 2016). Therefore, the current research will focus on children and young people with a diagnosis of ASD to identify changes in behaviour, skills, anxiety and also changes in parental stress and anxiety, since the introduction of COVID-19 restrictions in March 2020.

### Relaxation of COVID-19 restrictions

As Ireland progresses through the proposed Government road map and related phases, it is important to also address the new challenges which are emerging for children and their families, as restrictions continue to evolve. This will include working with children, parents, teachers and other professions to address the indirect impacts of COVID-19, specifically those that are felt with the easing of restrictions (and potentially their reintroduction in the coming weeks/months).

### Transition resource development

During the height of the COVID-19 restrictions in Ireland, an overwhelming number of resources were directed towards parents and children and young people which appeared to focus on dealing with the restrictions and their immediate impact. Little if any of these resources have considered the long-term impact and the need for support, particularly as restrictions have begun to ease in recent months.

In June 2020, the Department of Education announced the availability of Summer Provision 2020 to support families and children with a range of additional needs, including ASD. The programme could be run in either a school or home-setting for a minimum of 2 weeks and a maximum of 4 weeks over the July/August period 2020. However, the running of such a programme is dependent on the availability of the child’s school and/or a suitable tutor to come to their home. According to statistics reported by the Department of Education and Skills (2020), 243 schools around Ireland committed to providing the summer programme. As of 11th July 2020, the Department of Education and Skills (2020) reported that 10,291 families registered for the home-based programme in respect of 11,012 children. Therefore, while some children were able to avail of Summer Provision 2020, a considerable number of children and their families did not (e.g. if their school did not register to participate and/or they could not find a tutor to come to their home). These children and families will require intensive support and resources to facilitate transitions during a period of continued uncertainty in the coming weeks and months. 

Therefore, the primary aim of the current research is to develop an innovative, interactive, freely accessible package that will aid individuals with ASD to transition through the lifting of COVID-19 restrictions, by providing resources for them, their families and educators. The purpose of the transition toolkit is to provide one point of access for resources which will aim to address both the short- and long-term challenges experienced by children with autism as a result of the COVID-19 pandemic and subsequent easing of restrictions. It will be developed with parents, experts, experts by experience (including adults with autism, parents and family members) input and will signpost resources where they are currently freely available but also create new resources. It will comprise an online resource organised by category. Research prior to the onset of the COVID-19 pandemic identified a need for assistive technologies for people with autism to help with scheduling and planning as well as communication ( O’Neill *et al.*, 2020). It will be interesting to see if these themes emerge in the analysis of current needs. The content will be determined through a process of expert consensus and the resource will be beta tested before circulation.

### Aims of the research

In summary, the aims of the current study are twofold. First, to develop a needs-based resource toolkit for children, families and professionals working with individuals with autism. This toolkit will be developed through consultation with parents, young adults with autism, experts and experts by experience. Second, the study will assess the educational, psychological, behavioural and social impacts of COVID-19 restrictions and subsequent easing of restrictions on children and their parents. These impacts will be assessed longitudinally, across three different time points (Present, six month and one year follow up).

## Protocol

### Design

A strength of the current research is that it has involved stakeholders from conception through to funding application and now data collection. Those stakeholders include autism advocacy, teachers, and the national council for special education. A steering committee established before data collection commenced also includes representation from a parent and the Middletown Centre for Autism. The importance of stakeholder involvement also extends to the development of resources which will be informed by the needs assessment conducted with both parents and other stakeholders.

**Figure 1.**
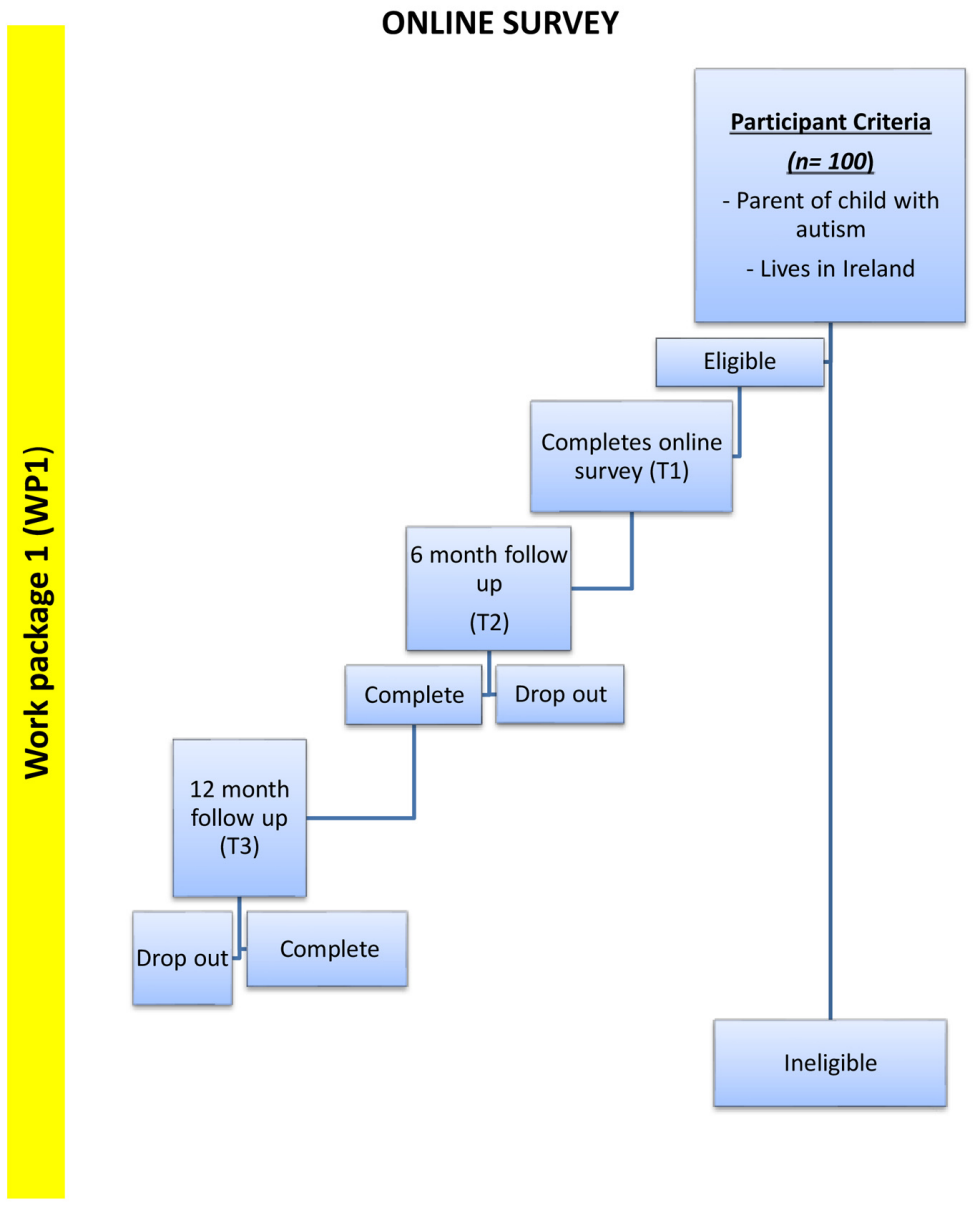
Flow chart depicting stages of Work Package 1 (WP1).

*Work package 1 (WP1)*: Will consist of a longitudinal study (N=100) measuring 1) parenting stress and 2) child’s levels of anxiety and challenging behaviour. This will be measured at Time 1 as early as possible in the restrictions, Time 2 (6 months) and Time 3 (12 months). The survey will also measure 3) current situation 4) resource needs and concerns for reintegration. It will inform the design Stage of Phase 3 resource development. Times 2 and 3 will inform on uptake and validity of the resource package. Participants will include parents of children (under 18) with a diagnosis of autism. It will also include the children themselves where their verbal abilities are such that they can respond to questions themselves (See Figure 1).

**Figure 2.**
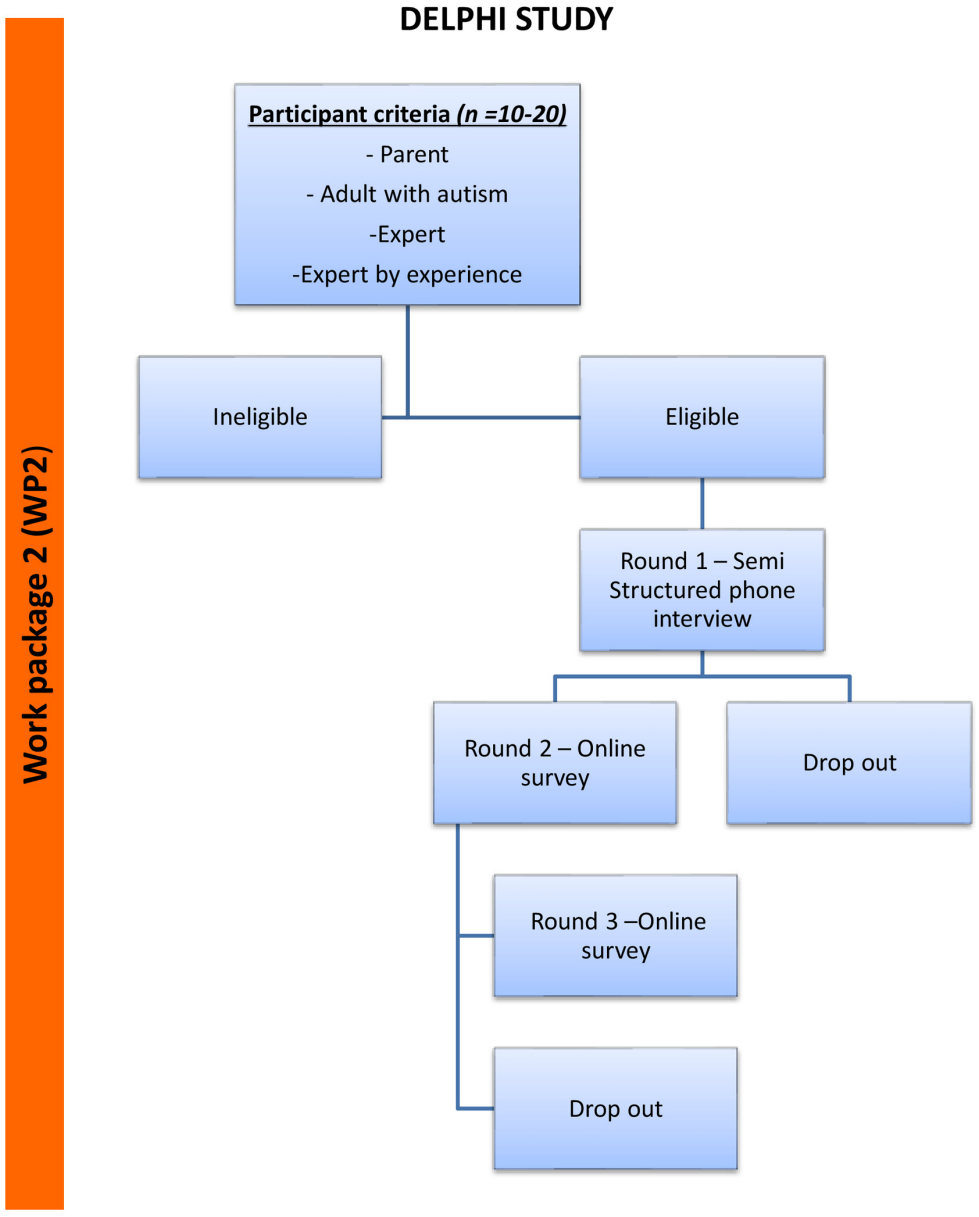
Flow chart depicting stages of Work Package 2 (WP2).

*Work package (WP2)*: In parallel to WP1, a Delphi study will achieve consensus on the challenges and facilitators to reintegration to a regular routine for children/young people with Autism Spectrum Disorder following the lifting of COVID-19 restrictions. Participants include stakeholders (e.g, young people with a diagnosis of ASD, parents, teachers, psychologists, therapists, and advocates). A minimum of 18 will be invited to take part in the proposed study to ensure each stakeholder group is sufficiently represented. Round 1 will be semi-structured interviews. After thematic analysis, a Round 2 questionnaire will be designed. In Round 2, respondents will be provided with a summary of some of the results from Round 1 and the WP1 survey. Participants will rank the areas of impact as well as the existing resources and desired resources in order of priority, flag potential interactions and add any barriers, motivators or strategies that should be included. The findings will inform the design of the resources in WP3. Long, medium- and short-term needs will be targeted (See Figure 2).

**Figure 3.**
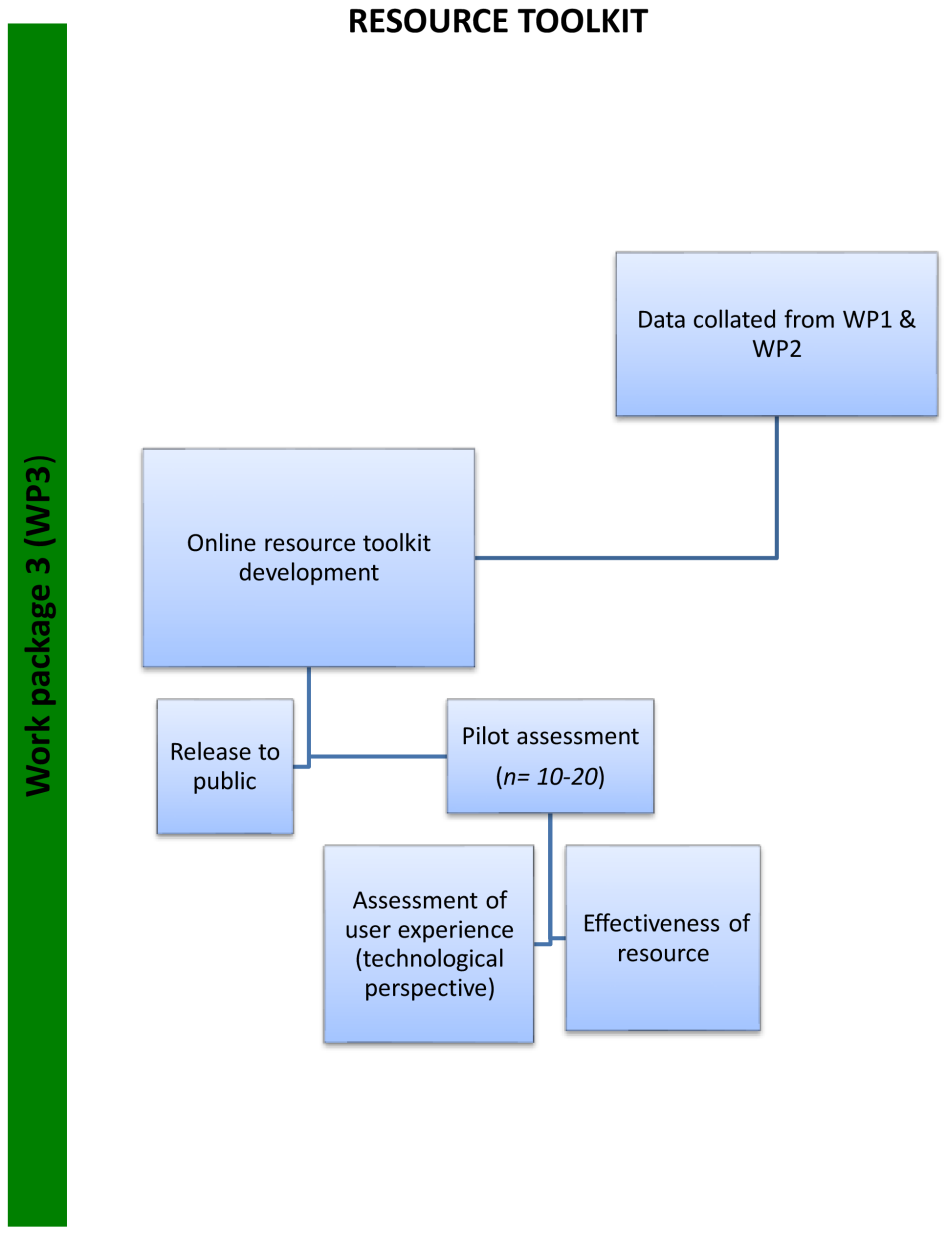
Flow chart depicting stages of Work Package 3 (WP3).

*Work package (WP3):* Development of an online transition package which will be available to children/young people with autism spectrum disorder, their parents/caregivers and educators, informed by WP1 and WP2. The design will be iterative with parents, educators and individuals with ASD and a pilot test will be conducted to assess resource effectiveness and usability (N=10–20). In line with previous research on the development of effective digital resources, Mummah *et al.* (2016) provide a comprehensive outline of the process from initial design to assessment and subsequent sharing of such resources. Both the assessment and simultaneous sharing of the resources developed in the current study will be grounded in the IDEAS (Integrate, Design, Assess and Share) process ( Mummah *et al.*, 2016). The mode and design of the package delivery will depend on WP2 recommendations. In keeping with existing interventions, use may be made of social stories, video modelling, teaching resources, training videos for parents and educators. This will be delivered through a custom designed user-friendly website (See Figure 3).

### Recruitment of study participants

*Work package 1 (WP1)*: Those who self-identify as a parent of a child (under 18) with a diagnosis of autism, living in Ireland will be invited to take part in the longitudinal online survey. Participants will be recruited through a number of means. Firstly, emails will be circulated to schools, advocacy groups and other relevant organisations with a request to circulate to their circles. Secondly, social media posts with a link to the survey will be prepared for LinkedIn, Facebook and Twitter and specific organisations and social media parent groups will be asked to share with their members. Finally, snowballing through social media and other means (e.g WhatsApp) will be utilized to ensure the survey is circulated as widely as possible.

**Work package 2 (WP2)**: Those who self-identify as a parent of a child with autism, a young adult with autism, an expert in the area of ASD (e.g. Psychologist, teacher, speech and language therapist, behavioural specialist etc.) and/or an expert by experience in ASD, will be invited to take part in the Delphi study. Similar to the recruitment of WP1, participants will be recruited through a variety of means. Invitation emails will be sent to relevant experts, experts by experience and autism advocates, identified by the PI and other collaborators on the research team. Social media posts with contact details for a member of the research team will be prepared and regularly circulated on Twitter and LinkedIn.

**Work package 3 (WP3):** Parents, children with autism, educators, therapists will be invited to pilot the online resources developed, providing feedback on their potential effectiveness and general usability of the platform (n = 10–20). Given the nature of the research, a convenience sample will be employed. Participants will be recruited through the online resource as well as through social media, media, emails to organisations, personal contacts and snowballing. 

### Sample size calculation

Prior experience of engaging in survey research with this population means that the PI has knowledge of the difficulty involved in encouraging engagement with surveys. This population in particular suffers from survey fatigue due to being the target of a large number of research studies prior to the advent of COVID-19. It is likely that this demand will increase due to interest in conducting research through a pandemic. Given these complicating factors and the exploratory nature of the research, a formal power analysis was not conducted; however, a sample size of 200 in Wave 1 of the survey is hoped for. In order to counter the possibility of low participant engagement with the WP1 survey, a Delphi study will be conducted as part of WP2 and this will focus not just on parents and individuals with autism but also on gaining expert opinion of experts and experts by experience, working with this population (e.g., teachers, psychologists, occupational therapists, speech and language therapists, parents etc.). There is no set criteria for sample size numbers in a Delphi study, however, there is a general agreement that the more panellists included, the greater the reliability of the group conclusions ( Santaguida *et al.*, 2018). Previous research varies in the number of participants from 15 participants ( Fiander & Burns, 1998) to 60 participants ( Alexander & Kroposki, 1999). In line with Delphi studies published recently therefore, a minimum of 18 participants will be recruited in order to ensure input from the main stakeholder groups ( Santaguida *et al.*, 2018). Finally, a sample of approximately 10 will be recruited in order to assess the toolkit. Previous research assessing toolkits and resources remains varied in their sample sizes. A recent study assessing the efficacy of a digitally-mediated social story for children with ASD recruited 15 participants ( Hanrahan *et al.*, 2020). Similarly, De Leo *et al.* (2011) assessed a smart-phone application for improving communication skills in children with ASD with three children and teachers. Therefore, a chosen sample size of 10 for the current study is in keeping with previous research in the area.

### Survey instrument

**Work package 1 (WP1):** The anonymous, online survey will comprise a combination of quantitative and qualitative items. Qualtrics software will be used to create the online survey (see extended data ( Smyth, 2021)). The survey items will cover areas such as: general demographics (e.g. age of participant, gender, work status, parenting relationship status, number of children, number of children with a diagnosis); a measure of parental stress; a measure of challenging behaviour; possible challenges anticipated for children; potential resources to address these challenges; and a standardized measure of child anxiety. Some measures and questions will remain consistent across times 1–3 while others may be added or substituted.

**Work package 2 (WP2):** The semi-structured interview for the Delphi study will use a combination of multiple choice and qualitative questions. The semi-structured interview will be conducted via phone with a research assistant and the participant, taking approximately 30–40 minutes. If the participant is not in a position and/or does not wish to participate via a phone interview, they will be invited to complete the questions via an online survey. The interview will consist of two sections. Section 1 will include a variety of demographic questions (e.g. participant age range, role/occupation, years of experience in ASD, years of experience in current role etc.). Section 2 of the interview will include open ended questions such as: both the short and long term difficulties faced by children, parents and professionals working in the area of ASD as a result of the COVID-19 easing of restrictions; possible resources and strategies to be used and/or created to lessen these challenges; and acceptability and use of online resources by children, parents and professionals. 

**Work package 3 (WP3):** The resource will be assessed through measurement of both the user experience from an information technology perspective and also the impact that it has had. This will be done through a series of questions that may be posed either through an online, paper or phone survey depending on the preference of the participants. It is anticipated that approximately 10–20 individuals will engage in an in depth assessment of the resource. This in-depth assessment will involve elements including interviews, ratings, and behaviour diaries where relevant. Metrics on the use of the resources will also be gathered via the online platform.

### Outcome measures

Primary outcome measures are the impact of the COVID-19 restrictions and subsequent easing of restrictions on children and young people with autism. These outcomes are explored generally through an online, anonymous survey and a semi structured interview, as part of a Delphi Study. However, specifically, these outcomes are measured using open ended questions in relation to the challenges anticipated for children and young people with autism in the coming months and the potential resources that could be created to lessen these impacts. Questions relating to challenging behaviours and anxiety levels are measured using a series of items developed by the research team and the anxiety scale for children with autism spectrum disorder (ASC-ASD) ( Rodgers *et al.*, 2016), respectively. Secondary outcomes of parental stress are also measured in the online survey using the Autism Parenting Stress Scale ( Silva & Schalock, 2012). Outcome measures for the Delphi study will be consensus regarding the challenges faced and the resources to prioritise. This will be measured through the rankings in the survey questions posed in Round 2 and any subsequent rounds of the Delphi study. Although there is a considerable body of literature devoted to the assessment of resources of various types many of which are similar to those in the toolkit ( De Leo *et al.*, 2011; Hanrahan *et al.*, 2020; Nicolaidis *et al.*, 2016), there is limited information about the assessment of a toolkit or collection of bespoke resources. Outcome measures for the toolkit assessment will include assessment of the use, usability and impact of the resource measured through survey, interview, behavioural diaries and measures of online activity on the resource platform. The measures may look at the toolkit as a whole or may focus on certain specific resources or collections of resources within it. If specific resources or resource collections are chosen for investigation, the selection of these will be informed by data from the WP2 Delphi study identifying the top ranked resources needed.

### Data analysis and statistical plan

Quantitative data included in the WP1 online survey will be analysed using IBM SPSS Statistical Software, Version 25. Both descriptive and frequency statistics will be conducted on quantitative data in order to determine the impacts of the current pandemic across time. Qualitative data collected in the online survey will be analysed using content analysis techniques. A similar process will be followed for the qualitative and quantitative data collected during the WP3 resource assessment. The main difference for WP3 will be that the small sample size may mean that the quantitative analysis is mainly descriptive or non-parametric depending on the exact participant numbers.

Qualitative data collected in the WP2 Delphi study, semi structured interviews will be transcribed and analysed using content and thematic analysis. This will be used to inform subsequent rounds of the Delphi study which will, for the most part, comprise ordinal data analysed through inferential and descriptive statistics.

### Data management and storage

A data management plan and report is currently being developed by the study team. The Principal Investigator (Dr. Sinead Smyth) will have primary responsibility for the data collected in this study. The raw data collected from the surveys will be stored for a maximum of 5 years, following the completion of the study, after which it will be destroyed by the principal investigator. Anonymised datasets may be published or shared with other researchers and may not be destroyed. Any data containing contact information for communication with participants will be destroyed as soon as the study has ended. Data from the Delphi study interviews is securely stored on password protected files and will be deleted following transcription. All data collected will be stored on the DCU network (GoogleDrive) to ensure that project stakeholders will have access to the data in a secure manner. 

### Ethics

All study procedures have received full ethical approval from The Research Ethics Committee, Dublin City University. Informed consent will be obtained from all adult participants and adult consent and child assent from any minors (aged 8–18 taking part). To ensure participant confidentiality is maintained, all participants create their own unique participant code and all data will be stored under that code, separate from any identifiable details such as email addresses. without any participant names and/or identifiable details included.

The research team concluded that the risk posed by participation was greater than in everyday life but still low. It is acknowledged that the potentially sensitive nature of some of the questions included in the longitudinal, online survey for parents. Particularly questions exploring parental stress levels and challenges their child and family have faced due to the impact of the COVID-19 restrictions, which may be distressing for some individuals to reflect on. Therefore, contact details for national support services are provided to participants, should they wish to engage in these supports.

### Plans for dissemination

Study findings will be prepared and disseminated in accordance with the audience it is targeting. Results from the study will be written up in several forms including as peer reviewed journal articles, lay and executive reports for stakeholders. An executive report, summarizing the key qualitative findings and basic frequencies from Wave 1, Phase 1, of the longitudinal online survey was uploaded to the study’s dedicated website and circulated to relevant bodies and institutions. Findings were also adapted into outputs such as infographics, video content (animations) and disseminated on the relevant social media platforms (e.g. Twitter) in order to target the wider lay audience. Participants will also have access to the platform of resources compiled by the researchers based on data collected in the study. Individual results or feedback will not be given to participants and this will be made clear at the point of consent.

### Study status

The study commenced data collection for Wave 1 of the survey on 25th June 2020 and Round 1 of the Delphi Study on the 17th July 2020. Round 2 of the Delphi Study is currently under way.

## Conclusion

The necessity for this research comes from the high prevalence of ASD in the Irish (and international) population and the impacts of COVID-19 and the resulting resource needs. Resources to help with school transitions, changes in routine or new experiences is not a new idea; however, never have such resources been needed on such a large scale (with every young person with ASD in the state affected simultaneously) or with such diversity (many activities not just school going will be affected). At the time at which the proposal for funding was written, the roadmap for restriction relaxation had not yet been written. The Department of Education has since announced the planned reopening of schools, for all children in August/September 2020, however, the delayed implementation of Phase 4 reopens, due to increases in positive COVID-19 cases, means that we must also prepare for possible school closures on a local and national level. The development of this resource package is therefore essential for the wellbeing of children and young people with autism, their families and teachers. It should be noted that resources will not be school specific but will also address reintroduction to regular activities which will have been suspended or which will now look very different than they did prior to the introduction of restrictions (e.g., going to the supermarket, for a haircut, a healthcare check-up, taking public transport, social encounters). The study is designed to minimise the detrimental psychological, social, educational and financial effects of the current COVID-19 crisis for a population which is demonstrably struggling at this time ( O’Brien, 2020).

## Data availability

### Underlying data

No data are associated with this article.

### Extended data

Figshare: T-Res Wave 1 survey questions. https://doi.org/10.6084/m9.figshare.13554353.v1 ( Smyth, 2021)

This project contains the following extended data:

- T-Res T1 survey.pdf (Study survey)

Data are available under the terms of the Creative Commons Attribution 4.0 International license (CC-BY 4.0).
