# Supplementary material for: Study protocol for autism specific transition resources (T-Res Study): developing a flexible resource package for dealing with the loosening and/or lifting of COVID-19 related restrictions
Source: HRB Open Res. 2021 Jan 28;4:12. [Version 1] doi: 10.12688/hrbopenres.13155.1 (PMC8686326; doi:10.12688/hrbopenres.13155.1)
Supplement: Peer review history

# Peer review: Reviewer response for version 1

**DOI:** 10.21956/hrbopenres.14275.r31037

**Published:** 2021-12-17

**Authors:** David Marshall

**Affiliations:** 1 Centre for Reviews and Dissemination, University of York, York, UK

This protocol attempts to address an issue which is of serious concern to the future of the autistic community. I agree with the authors that it is proposed research is vital to consider the long-term impact and the need for support in the face of COVID restrictions. It is particularly timely and shows a great deal of proactive thinking to plan for how to support these young people during the period where these restriction may be loosened. The authors' aims are particularly pertinent but also potentially adaptable in that they are to first develop a needs-based resource toolkit and secondly to track family experiences of young people with autism during this stage of the pandemic. 

 The arguments for this study are well made and the structural integrity of the paper is excellent. I have two minor issues with the methods employed but overall I feel this section is also very strong. First, on the measures used, the authors have not been completely clear how they are collecting what the restrictions are in the intervening periods of the study and how this will be translated into their impact. The one thing that is most clear about our experiences of the pandemic is that it is unpredictable and our route out will likely be bumpy. It may also be the case that different communities may have different levels of restrictions at the same timepoint. Will the authors have questions which ask the respondents what the current restrictions are as well as how the restrictions in general are impacting on their lives? Second, I agree with the previous reviewer that there is significant scope for expanding on the analysis section. I would also add that the quantitative also needs some detail as it currently gives the reader no sense of what the data will be used for. The section really stand in contrast to the rest of the paper which is excellent so I hope the authors will give us a clearer picture of their plans.

Is the study design appropriate for the research question?

Yes

Is the rationale for, and objectives of, the study clearly described?

Yes

Are sufficient details of the methods provided to allow replication by others?

Yes

Are the datasets clearly presented in a useable and accessible format?

Not applicable

Reviewer Expertise:

Autism Spectrum Disorders and Systematic Reviews

I confirm that I have read this submission and believe that I have an appropriate level of expertise to confirm that it is of an acceptable scientific standard.

# Peer review: Reviewer response for version 1

**DOI:** 10.21956/hrbopenres.14275.r29181

**Published:** 2021-04-27

**Authors:** Jennifer McMahon

**Affiliations:** 1 Department of Psychology, University of Limerick, Limerick, Ireland

The proposed research is set against a backdrop of the needs of students with autism during the easing of COVID-19 restrictions. Young people with autism have been one of the groups most affected by the pandemic and so the proposed research is timely and necessary. The aim of the research is two-fold, the first is to develop a needs-based resource toolkit for children, families and professionals working with children with autism. The second is to track family experiences of young people with autism across the pandemic. 

 The protocol is well written and has most of the important information. However the data analysis and statistical plan section could be strengthened. Please discuss any possible inferential statistics that will be employed and handling of data. Please also stipulate the framework for the qualitative analysis and provide additional detail on the iterative process to be employed in the Delphi study.

Is the study design appropriate for the research question?

Yes

Is the rationale for, and objectives of, the study clearly described?

Yes

Are sufficient details of the methods provided to allow replication by others?

Yes

Are the datasets clearly presented in a useable and accessible format?

Not applicable

Reviewer Expertise:

Autism, evidence based practice, implementation science, special education, mental health

I confirm that I have read this submission and believe that I have an appropriate level of expertise to confirm that it is of an acceptable scientific standard.
